# Integration of transcriptomic and proteomic analyses reveals several levels of metabolic regulation in the excess starch and early senescent leaf mutant *lses1* in rice

**DOI:** 10.1186/s12870-022-03510-2

**Published:** 2022-03-23

**Authors:** Zhiming Chen, Yongsheng Wang, Rongyu Huang, Zesen Zhang, Jinpeng Huang, Feng Yu, Yaohai Lin, Yuchun Guo, Kangjing Liang, Yuanchang Zhou, Fangyu Chen

**Affiliations:** 1 Key Laboratory of Ministry of Education for Genetic Improvement and Comprehensive Utilization of Crops, Fujian Provincial Key Laboratory of Crop Breeding by Design, College of Agriculture, Fujian Agriculture and Forestry University, Fuzhou, 350002 Fujian China; 2 Postdoctoral Station of Biology, School of Life Sciences, Hebei University, Baoding, 071000 Hebei China; 3 School of Life Sciences, Xiamen University, Xiamen, 361005 Fujian China; 4 College of Computer and Information Science, Fujian Agriculture and Forestry University, Fuzhou, 350002 Fujian China

**Keywords:** Transcriptome, Proteome, *lses1*, Leaf senescence, Transitory starch, Rice (*Oryza sativa* L.)

## Abstract

**Background:**

The normal metabolism of transitory starch in leaves plays an important role in ensuring photosynthesis, delaying senescence and maintaining high yield in crops. OsCKI1 (casein kinase I1) plays crucial regulatory roles in multiple important physiological processes, including root development, hormonal signaling and low temperature-treatment adaptive growth in rice; however, its potential role in regulating temporary starch metabolism or premature leaf senescence remains unclear. To reveal the molecular regulatory mechanism of *OsCKI1* in rice leaves, physiological, transcriptomic and proteomic analyses of leaves of *osckI1* allele mutant *lses1* (*leaf starch excess and senescence 1*) and its wild-type varieties (WT) were performed.

**Results:**

Phenotypic identification and physiological measurements showed that the *lses1* mutant exhibited starch excess in the leaves and an obvious leaf tip withering phenotype as well as high ROS and MDA contents, low chlorophyll content and protective enzyme activities compared to WT. The correlation analyses between protein and mRNA abundance are weak or limited. However, the changes of several important genes related to carbohydrate metabolism and apoptosis at the mRNA and protein levels were consistent. The protein-protein interaction (PPI) network might play accessory roles in promoting premature senescence of *lses1* leaves. Comprehensive transcriptomic and proteomic analysis indicated that multiple key genes/proteins related to starch and sugar metabolism, apoptosis and ABA signaling exhibited significant differential expression. Abnormal increase in temporary starch was highly correlated with the expression of starch biosynthesis-related genes, which might be the main factor that causes premature leaf senescence and changes in multiple metabolic levels in leaves of *lses1*. In addition, four proteins associated with ABA accumulation and signaling, and three CKI potential target proteins related to starch biosynthesis were up-regulated in the *lses1* mutant, suggesting that *LSES1* may affect temporary starch accumulation and premature leaf senescence through phosphorylation crosstalk ABA signaling and starch anabolic pathways.

**Conclusion:**

The current study established the high correlation between the changes in physiological characteristics and mRNA and protein expression profiles in *lses1* leaves, and emphasized the positive effect of excessive starch on accelerating premature leaf senescence. The expression patterns of genes/proteins related to starch biosynthesis and ABA signaling were analyzed via transcriptomes and proteomes, which provided a novel direction and research basis for the subsequent exploration of the regulation mechanism of temporary starch and apoptosis via *LSES1*/*OsCKI1* in rice*.*

**Supplementary Information:**

The online version contains supplementary material available at 10.1186/s12870-022-03510-2.

## Background

Leaves are a critical metabolic source for plants to photosynthesize and produce organic energy materials, and the developmental status of leaves is closely related to the yield of crops. Starch, which includes storage starch in storage organs and transitory starch in photosynthetic organs, is the predominant carbohydrate synthesized by photosynthesis. It not only plays an important role in growth, development and reproduction in plants but also serves as the most important food, feed and energy source for human beings [1]. Therefore, the regulatory mechanism of starch synthesis, degradation and accumulation has become a research focus in plant physiology. In leaves, transitory starch is formed in chloroplasts during the day and broken down hydrolytically and phosphorolytically at night [2]. Using forward and reverse genetics, more than 40 enzymes have been discovered that are active in the process of transitory starch turnover in *Arabidopsis* [3]. However, the underlying mechanism with details of transitory starch turnover is presently poorly understood.

In the postgenomic era, the iTRAQ (isobaric tags for relative and absolute quantification)-based quantitative proteomics approach is an important large-scale, high-throughput analysis tool for investigating biological systems [4–6]. Precise analysis of the proteome is essential for understanding the regulation of transitory starch, which needs to be further elucidated at multiple levels. Genome-wide proteomic analyses are crucial for providing accurate pictures of the regulatory networks of functional genes and proteins. However, how changes in transcriptional, posttranscriptional or even translational control are reflected in changes at the protein level remains unclear. Poor correlations between mRNA and protein expression have often been reported [7, 8]. A recent study in a fibreless mutant of cotton using integrative transcriptome, proteome, phosphoproteome and genetic mapping emphasized the importance of the translational regulation of protein abundance [9], reflecting the significance of a comprehensive view of gene expression.

*OsCKI1*, which is known as *hbd2/LTRPK1/LTG1*, encodes a member of the casein kinase I family and has been reported to be involved in hybrid breakdown, root development, hormone response and cold adaptation [10–13]. *EL1* is a homologous gene of *OsCKI1*, which phosphorylates the stability of DELLA protein Slender Rice 1 (SLR1) to inhibit gibberellin signaling and rice heading [14]. In Arabidopsis (*Arabidopsis thaliana*), the latest research shows that plant CK1 can regulate cell cycle/division by modulating the stability and inhibition of cyclin-dependent kinase inhibitor 6 (KRP6) through phosphorylation [15]. CK1-like 6 (CKL6) phosphorylates and binds to cortical tubules to regulate cell growth and morphogenesis [16], while CK1.3 and CK1.4 phosphorylate the blue light receptor cryptochrome 2 (CRY2) to regulate blue light-mediated photomorphogenesis [17]. In addition, CK1 is closely related to endogenous hormone response in plant and phosphorylation plays a major role [11, 14, 17, 18]. Chen et al. (2018) has reported that CKI phosphorylates PYR/PYLs to promote its ubiquitination and degradation, thereby inhibiting the ABA response [18], suggesting that proteins or enzymes that mediates the function of ABA may act as a substrate of CKI, allowing to participate in the ABA response [11]. Min et al. (2013) have reported that CKI exhibits starch synthase kinase activity, increases glucose concentration in young buds and activates ABA accumulation, thereby disturbing the balance of reactive oxygen species and ultimately destroying the tapetum PCD, leading to anther inactivation [19]. To date, the roles of CKI in plant growth and development have been extensively researched, but no results have linked *OsCKI1* to regulating temporary starch metabolism or premature leaf senescence in rice.

Here, we report an integrative analysis of the transcriptome and proteome in an *OsCKI1*-deficient allelic mutant *lses1* (*leaf starch excess and senescence 1*), which has an obvious premature senescence and excess starch phenotype in leaves. The physiological characteristics and differentially expressed genes/proteins in *lses1* were analysed. The results indicated that several key functional categories, such as carbohydrate metabolism, response to hormone stimulus, pigment metabolism, translation, cytoplasmic part and plastid organization, were significantly enriched. Moreover, it was found that changes in the physiological characteristics of leaves were directly reflected in the mRNA and protein levels, which were consistent with the physiological measurement results and the omics data. Notably, this result also suggested that ABA (abscisic acid) may be involved in multiple metabolic regulation via *LSES1*/*OsCKI1* and the formation of mutant phenotypes in *lses1* leaves. This result may be beneficial to the study of the genetic regulation of temporary starch in rice leaves.

## Methods

### Plant materials and growing conditions

The stably inherited mutant *lses1* (*leaf starch excess and senescence 1*) with an obvious leaf tip withering phenotype was derived from the progeny of rice *indica* restorer line Hanhui1014 seeds treated with γ-ray irradiation. Hanhui1014 plants represent the wild-type (WT). Our early genetic analysis showed that the ratio between F_2_ normal plants and mutant plants of the cross combination of *lses1* and multiple varieties was 3:1, indicating the phenotype of *lses1* was controlled by a recessive nuclear gene. Further gene fine-mapping and sequencing results showed that *LSES1* was located to the long arm of rice chromosome 2 and is allelic to *OsCKI1* (LOC_Os02g40860), which encodes a CKI protein, also known as *hbd2* [10], *LTRPK1* [11, 12] and *LTG1* [13]. Similar to the mutant *ltg1* [13], a T to A missense mutation at nucleotide 1070 in the coding region resulted in the amino acid substitution of isoleucine to lysine (I357K) in the LSES1 protein in mutant *lses1* (our unpublished data). All the plant materials were provided by the Laboratory of Germplasm Resources Innovation and Utilization, Fujian Agriculture and Forestry University. Rice plants were cultivated under natural conditions in a paddy field Fujian Agriculture and Forestry University. Pregerminated seeds were sown, and the seedlings were transplanted with an interplant spacing of 20 × 20 cm^2^.

### Determination of starch content and I_2_-KI staining in leaves

The phenotype of excessive starch and premature senescence of *lses1* leaves appeared from three-leaf stage to tillering stage. Therefore, the leaves of WT and *lses1* at six-leaf stage which is the transition period between seedling stage and tillering stage were used for the following analyses. The second leaf from the top of WT and *lses1* seedlings at the sixth leaf stage was harvested at the end of the night (6:00) and at the end of the day (18:00), frozen in liquid nitrogen and stored at − 80 °C. Approximately 0.2 g frozen leaf samples were ground using a mortar and pestle under cryogenic conditions. The leaf powder was measured by using a starch content assay kit (BC0700, Solarbio, China) according to the manufacturer’s assay procedure. At the same time, the first, second, and third leaves from the top of the WT and *lses1* seedlings were sampled and stained with I_2_-KI solution as described by Hagen et al. (2008) with minor modifications [20]. The leaves were incubated in an acetone/ethanol (1:1, v/v) mixture and placed at room temperature for 24-36 h until the chlorophyll was removed. After bleaching, the leaves were put in I_2_-KI solution for approximately 20 min and then removed and placed on a table. Photographs were taken and saved with a camera. Three biological replicates of each sample were performed.

### Determination of photosynthetic pigment content

At 8:30 on a sunny day when photosynthesis has started and entered the photosynthetic state, the second leaf from the top of WT and *lses1* seedlings at the sixth leaf stage was harvested. Measurement of the photosynthetic pigment content was performed according to the procedures of Wellburn (1994) with slight modification [21]. Briefly, approximately 0.1 g of cut leaves was soaked in a centrifuge tube with a 25 mL mixed solution of acetone/absolute ethanol (1:1, v/v). After 24 h in the dark, the absorbance values of the soaking liquid at wavelengths of 663 nm, 645 nm and 470 nm were measured separately by using a spectrophotometer (Beckman Coulter-DU720, USA). Then, the contents of Chl *a*, Chl *b* and total Chl were calculated by referring to standard formulas. Three biological replicates of each sample were performed.

### Determination of lipid peroxidation and ROS-scavenging enzymes

The second leaf from the top of WT and *lses1* seedlings at the sixth leaf stage was sampled at 8:30 on a sunny day and quickly frozen in liquid nitrogen before storage at − 80 °C. The weighed frozen leaf samples were ground on ice by using a mortar and pestle containing phosphate buffer. After grinding, the homogenate was used to determine the contents of MDA, H_2_O_2_, O_2_- and •OH and the activities of CAT, SOD and POD according to the methods of kits purchased from the Nanjing Jiancheng Bioengineering Institute (China). The kits were A003-1, A064, A052, A018, A007-1, A001-1 and A084-3, respectively. Three biological replicates of each sample were performed.

### RNA-seq assays and data analysis

At the end of the night (6:00) when the difference in the temporary starch degradation and metabolism status of WT and *lses1* leaves is most obvious, ten-centimetre-long segments of the leaf tip from the second leaf from the top of seedlings at the sixth leaf stage were harvested, and quickly frozen in liquid nitrogen and stored at − 80 °C. Three biological replicates of each sample were performed, and each replicate contained ten leaf segments. RNA-seq analysis was performed by Annoroad Gene Technology (Beijing, China). Total RNA was extracted from leaves with TRIzol reagent (Invitrogen) following the manufacturer’s instructions. The RNA concentration was measured with a Nanodrop spectrophotometer (Thermo), and RNA integrity was checked using a 1% agarose gel and an Agilent 2100 Bioanalyzer. After poly(A) mRNA isolation, cDNA libraries for RNA-seq were constructed using an Illumina gene expression sample prep kit (Illumina). Deep sequencing of the cDNA libraries was accomplished by Annoroad Genome. The reference genome information was from the Rice Genome Annotation Project (http://rice.plantbiology.msu.edu). The total reads of every gene locus were counted using ERANGE software (http://woldlab.caltech.edu/gitweb/). The expression level of each gene was normalized to the RPKM (reads per kilobases per million reads) value.

### Total protein extraction, digestion and iTRAQ labeling

The leaf samples used for iTRAQ analysis were the same as those used for RNA-seq analysis. Protein extraction and digestion of each sample for proteomic analysis were performed according to Han et al. (2014) with minor modifications [22]. Briefly, the digested sample was desalted using a Sep-Pak C18 column (Waters, Milford, MA, USA). Sample was firstly washed with 300 μL Milli-Q water, subsequently eluted in 50 μL methyl alcohol, and finally dried under vacuum and stored at − 80 °C for further use. iTRAQ labelling of peptides was performed using an iTRAQ 4-plex kit (Applied Biosystems, USA) according to the manufacturer’s instructions, dried by vacuum centrifugation and stored at − 80 °C until LC-MS/MS analysis.

### LC-MS/MS analysis

Samples were analysed on a nanoElute (plug-in V1.1.0.27; Bruker, Bremen, Germany) coupled to a timsTOF Pro (Bruker, Bremen, Germany) equipped with a CaptiveSpray source. All raw files were analysed by Peaks Studio X software (Bioinformatics Solutions Inc., Waterloo, ON, Canada). Data were searched against the rice UniProt Reference Proteome with isoforms (downloaded December 2019). De novo sequencing of peptides, database searching and characterization of specific PTMs were performed to analyse the raw data; the false discovery rate (FDR) was set to ≤1%, and [− 10*log(p)] was calculated accordingly, where p is the probability that an observed match is a random event.

### Bioinformatics analysis

The differentially expressed proteins (DEPs) and genes (DEGs) between WT and *lses1* were identified by using the DESeq R package [23]. Proteins with a fold change greater than 1.5 were considered DEPs. The genes that had a fold change greater than 2.0 and a *p*-value < 0.05 were regarded as DEGs. The hierarchical cluster analysis of DEGs according to the gene expression pattern in each sample was performed utilizing the pheatmap package in R [24]. Gene Ontology (GO) enrichment analyses of DEGs and DEPs were performed by using the BiNGO plugin in Cytoscape based on the parameter FDR = 0.05 [25]. The protein-protein interactions in rice were assessed by the STRING database (http://string-db.org/), and the functional clusters of the protein-protein interaction network containing DEPs were distinguished by the MCODE [26] plugin in Cytoscape with built-in parameters.

## Results

### Phenotypic and physiological characteristics of *lses1* leaves

**Fig. 1:**
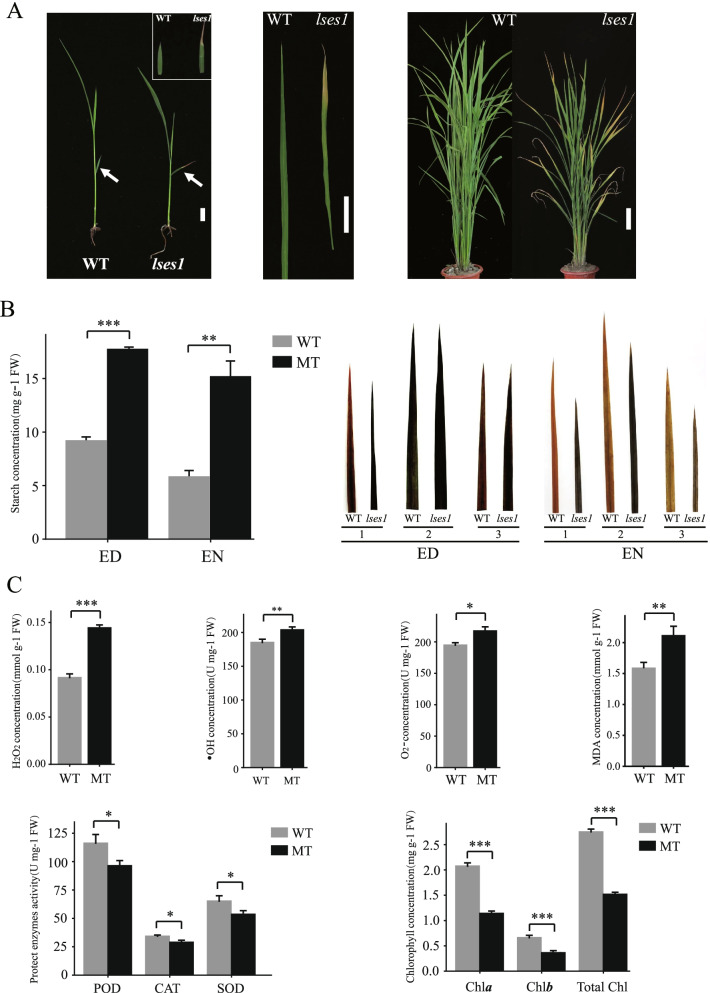
Phenotypes and physiological characteristics of the wild type (WT) and *lses1* mutant. **A** Phenotypes of the plants and leaves of WT and *lses1* at the different growth stages. Phenotypes of the plants of WT and *lses1* at the third-leaf stage (left), scale bar is 1 cm; Phenotypes of the second leaves from the top of WT and *lses1* seedlings at the sixth-leaf stage (mid), scale bar is 5 cm; Phenotypes of the plants of WT and *lses1* at the tillering stage (right), scale bar is 10 cm. **B** Starch contents (left) and I_2_-KI staining (right) of the leaves of WT and *lses1*. ED, sampling at the end of the day; EN, sampling at the end of the night. 1, 2 and 3 represent the first, second and third leaves from the top of seedlings at the sixth leaf stage, respectively. **C** Contents of ROS and chlorophyll and activities of protective enzymes in the leaves of wild-type (WT) and *lses1* mutant (MT) plants. Error bars represent the standard deviations of three biological replicates. ^*^, ^**^ and ^***^ significant difference at *P* < 0.05, *P* < 0.01 and *P* < 0.001, respectively

No phenotypic differences between the *lses1* mutant and WT were observed before the three-leaf stage. However, compared with WT plants, the older leaf blades of *lses1* started to wither at the leaf tips from the third-leaf stage and began to exhibit a premature leaf senescence phenotype (Fig. 1-A left), and the withering degree deepened with the development process and gradually developed at higher leaf positions (Fig. 1-A mid and right). The starch contents in leaves were determined at 6:00 (the end of night, EN) and 18:00 (the end of day, ED); the values of *lses1* at 18:00 and 6:00 were 1.9 and 2.7 times that of WT, respectively, and the value at 6:00 was slightly lower than that at 18:00 in *lses1* (Fig. 1-B left and Table S1). In the same period, seedlings of the *lses1* mutant and WT were stained with I_2_-KI solution. At 18:00, the leaves of the three leaf positions in *lses1* and WT were dyed dark blue by I_2_-KI solution, but the colour of *lses1* leaves was slightly darker. At 6:00, the colour of WT leaves was pale yellowish brown, while the leaves of *lses1* were still blue to varying degrees (Fig. 1-B right). Taken together, the results indicated that the starch produced by photosynthesis in the leaves of *lses1* during the day was incompletely decomposed and utilized at night.

Furthermore, determinations of other physiological characteristics were also performed in the leaves of *lses1* and WT at the seedling stage. The results showed that the contents of H_2_O_2_, O_2_-, •OH and MDA of *lses1* leaves were significantly higher than those of WT leaves to varying degrees (Fig. 1-C top and Table S1), while the activities of SOD, POD and CAT were significantly lower in *lses1* than in WT (Fig. 1-C bottom-left and Table S1). The Chl *a*, Chl *b* and total Chl contents in *lses1* leaves were all significantly lower than those in WT leaves (Fig. 1-C bottom-right and Table S1). These results implied that increased lipid peroxidation, an imbalance of ROS-scavenging systems and chlorophyll degradation occurred in *lses1* leaves.

### Identification and analysis of DEGs

**Fig. 2:**
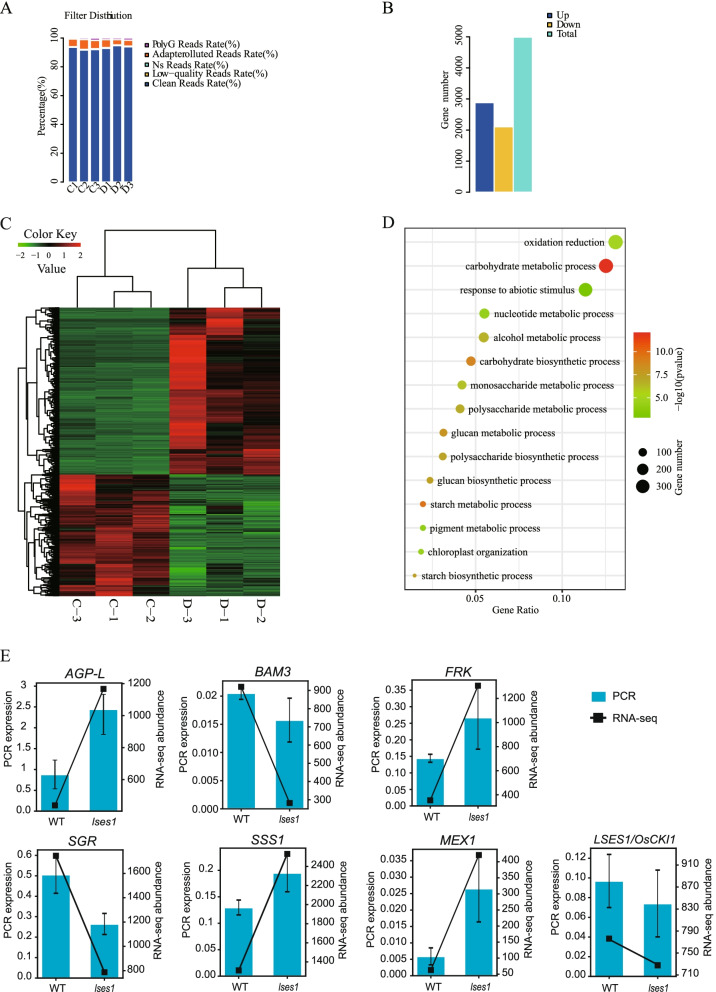
Identification and analysis of DEGs (differentially expressed genes) expressed in clusters between the wild type (WT) and the *lses1* mutant. **A** The ratio of reads in the transcriptome. C1-C3, wild-type repeats 1-3; D1-D3, *lses1* mutant repeats 1-3. **B** Statistical results of the DEGs. **C** Heatmap of the DEGs. C1-C3, wild-type repeats 1-3; D1-D3, *lses1* mutant repeats 1-3; I and II represent clusters I and II with similar gene expression patterns, respectively. **D** GO-BP enrichment analysis of DEGs from the two clusters. The top 15 GO categories of each cluster are shown. **E** The expression levels of six DEGs and *LSES1* in RNA-seq and RT-qPCR

To obtain more information at the mRNA level, a comparative transcriptomic analysis of *lses1* and WT leaves was performed. Sequencing quality evaluation showed that the clean reads rate of each sample after filtering exceeds 90% (Fig. 2-A and Table S2), indicating that these results were sufficient for subsequent analysis. As shown in Fig. 2-B and Table S3, 4989 (2883 upregulated and 2106 downregulated) differentially expressed genes (DEGs) were identified between *lses1* and WT with the following parameters: *p*-value < 0.05 and fold change > 2 (Fig. 2-B and Table S3), and these DEGs were congregated into two distinct modules according to their expression level in each sample (Fig. 2-C). GO-BP enrichment analysis results suggested that several apoptosis and starch metabolism-related categories were significantly enriched, including carbohydrate metabolic process, oxidation reduction, starch metabolic process, response to abiotic stimulus, nucleotide metabolic process, alcohol metabolic process and pigment metabolic process (Fig. 2-D). To validate the transcriptome data, expression levels of seven transcripts were detected using RT-qPCR, and the results were compared with RNA-seq data. As shown in Fig. 2-E, expression trends of seven transcripts determined in RT-qPCR is consistent with that in RNA-seq, indicating the reliability of RNA-seq data. Interestingly, there is no significant difference in the expression of *LSES1* between WT and *lses1* mutant (Fig. 2-E), which implied that single-base missense mutation of *LSES1* did not affect transcript accumulation.

### Identification and analysis of DEPs

**Fig. 3:**
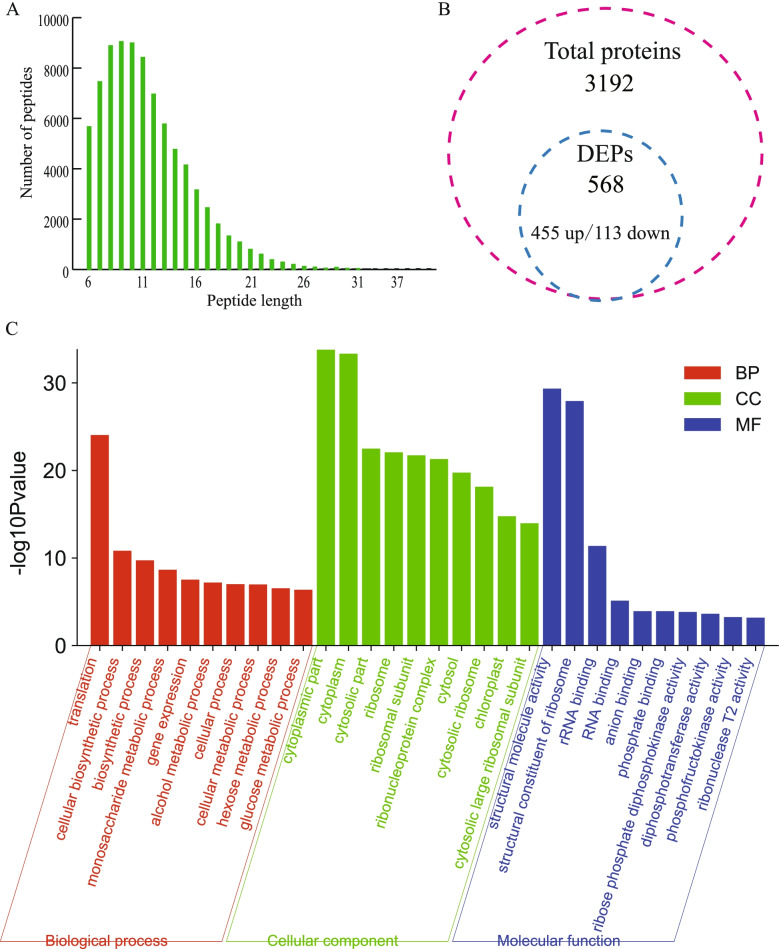
Identification and analysis of DEPs (differentially expressed proteins) between the wild type (WT) and the *lses1* mutant*. ***A** Distribution of peptide length and number in the whole proteome. **B** Statistical results of the DEPs. C, GO enrichment analysis of the DEPs. The top 10 GO functional subcategories of the three main categories are shown

Proteome profile analyses were performed on the leaves of *lses1* and WT. A total of 82,903 peptides were detected, and the lengths of peptides, as determined by proteomics, ranged mostly from 6 to 12 aa (Fig. 3-A). Of these peptides, 3192 proteins contained at least two unique peptides were identified, including 568 (455 up-regulated and 113 down-regulated) differentially expressed proteins (DEPs) between *lses1* and WT (Fig. 3-B and Table S4). As expected, the LSES1 protein was not found to be expressed in *lses1* mutant, which suggested that the amino acid substitution due to single-base missense mutation of *LSES1* might change the protein structure. We subsequently performed the GO enrichment analysis of these 568 DEPs. As shown in Fig. 3-C, in the biological process category, translation was the most enriched subclass, followed by cellular biosynthetic process, biosynthetic process, and monosaccharide metabolic process. In the cellular component category, cytoplasmic part was the subclass with the highest enrichment score, followed by cytoplasm, cytosolic part, and ribosome. In the molecular function category, structural molecule activity occupied the top place, followed by structural constituent of the ribosome, rRNA binding, and RNA binding.

### Functional clusters in the PPI network

**Fig. 4:**
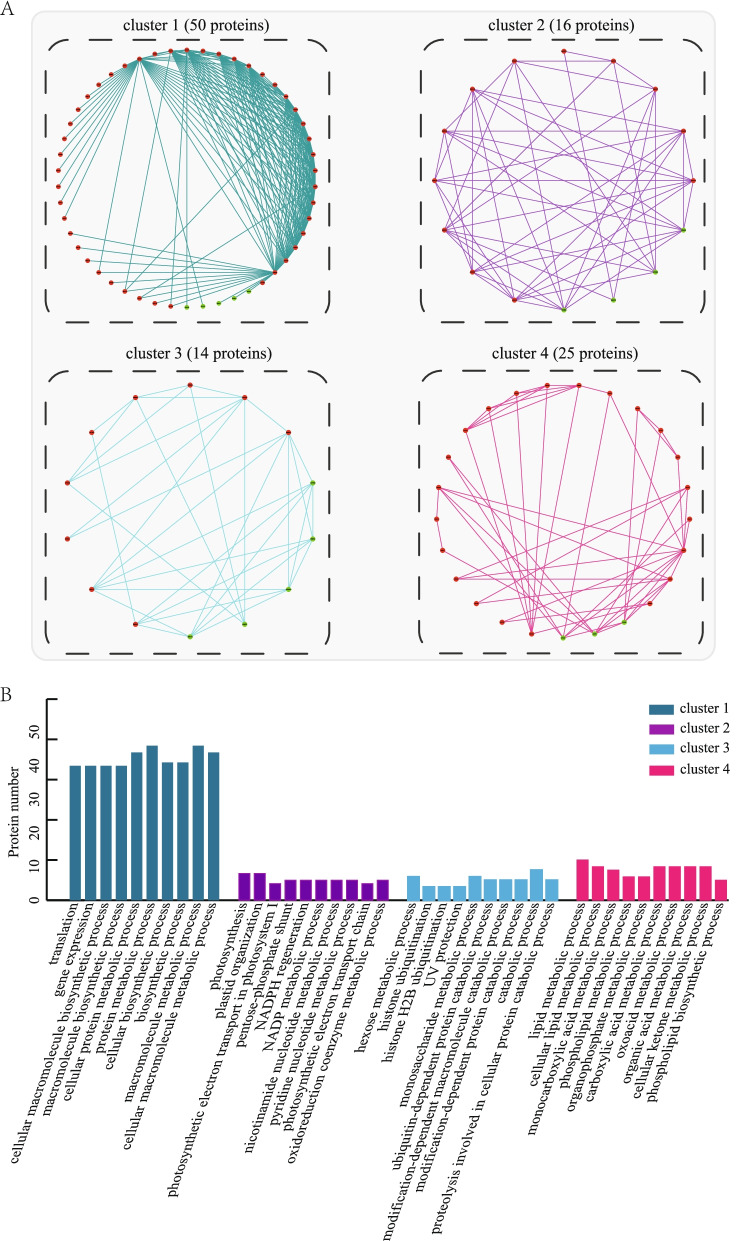
Functional cluster analyses of the PPI (protein-protein interaction) network. **A** Four functional clusters distinguished from the PPI network. Red dots represent upregulated proteins, and green dots represent downregulated proteins. **B** GO-BP enrichment analyses of proteins in each of 4 clusters. The bar graph shows the top 10 GO categories in each cluster

To explore the biological characteristics of the 568 differentially expressed proteins, a protein-protein interaction (PPI) network was constructed by integrating these proteins and the known STRING interactions and distinguishing the functional clusters via the MCODE [18] plugin for Cytoscape according to the default parameters. In total, four highly related functional clusters were identified and named clusters 1, 2, 3 and 4, which included 50, 16, 14 and 25 proteins, respectively (Fig. 4-A and Table S5). Notably, two sugar metabolism-related proteins glucose-6-phosphate 1-dehydrogenase (A2YKG1) and ATP-dependent 6-phosphofructokinase (A2WLL3) and four cell proliferation-related proteins eukaryotic translation initiation factor (A2XZF9, B8AZD7, B8ACZ5 and B8AE20) and two phospholipid metabolism-related proteins phosphatidylserine decarboxylase proenzyme 1 (B8AL07) and phospholipase D (Q9LKM2) were found in the PPI network. GO-BP enrichment analysis of these four clusters was performed. As shown in Fig. 4-B, several key processes were significantly enriched in the four clusters: protein metabolic process, macromolecule metabolic process, photosynthesis, plastid organization, catabolic process, hexose metabolic process, lipid metabolic process and organic acid process. Overall, these results emphasize the potential roles of PPI network in contributing to the formation of excess starch and premature senescence phenotypes of *lses1* leaves.

### Carbohydrate metabolism- and apoptosis metabolism-associated genes/proteins

Thus far, comprehensive proteome and transcriptome studies have shown that the correlation between mRNA levels and protein expression across large data sets is generally weak [7, 8, 27]. In this study, about 20 % of detected proteins were regulated at the transcription level which was similar with previous researches. To more fully clarify the regulation mechanism of temporary starch and premature senescence of rice leaves, the differentially expressed genes/proteins related to carbohydrate metabolism and apoptosis metabolism were comprehensively screened from WT and *lses1* in the following analysis.

**Fig. 5:**
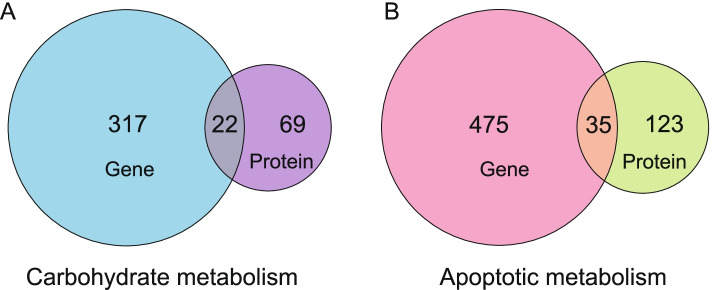
Statistics of differentially expressed proteins/genes related to carbohydrate and apoptotic metabolism. **A** The number of DEGs/DEPs related to carbohydrate metabolism. **B** The number of DEGs/DEPs related to apoptotic metabolism

339 DEGs and 91 DEPs involved in carbohydrate metabolism were subsequently identified between *lses1* and WT (Fig. 5-A and Table S6). As expected, several starch and sugar metabolism-related genes showed a consistent trend of changes at the mRNA and protein levels. For example, the genes encoding glucose-1-phosphate adenylyltransferase large subunit (AGP-L), chloroplastic maltose excess protein 1-like (MEX1), soluble starch synthase 1 (SSS1), sucrose synthase 1 (SUS1), fructokinase 2 (FRK-2), pyrophosphate--fructose 6-phosphate 1-phosphotransferase subunit alpha (PFP-ALPHA), cytosolic pyruvate kinase isozyme (PK), malate dehydrogenase (MDH), alcohol dehydrogenase 1 (ADH1) and trehalose-6-phosphate synthase 3 (TPS3) all exhibited increased transcripts and protein abundance (Table S6). Other genes/proteins in *lses1*, such as the genes encoding chloroplastic β-amylase 3 (BAM3) involved in starch metabolism, glyceraldehyde-3-phosphate dehydrogenase (GAPC) involved in glycolysis and trehalase (TRE) involved in trehalose metabolism also showed altered transcription levels (Table S6). Overall, the up- and down-regulation of these genes/proteins might lead to the imbalance of carbohydrate metabolism in *lses1* leaves.

Furthermore, 510 DEGs and 158 DEPs involved in apoptosis-related metabolism in *lses1* showed increased or decreased abundance (Fig. 5-B and Table S7). Notably, the genes encoding L-ascorbate peroxidase (APX1; APX3) involved in ROS scavenging, nonspecific lipid-transfer protein (nsLTP2) involved in phospholipid metabolism, and adenine phosphoribosyltransferase form 2 (APRT) involved in nucleic acid metabolism were regulated at both mRNA and protein levels (Table S7). Several genes/proteins associated with ROS production, such as the protein photosystem I iron-sulfur centre (psaC) and the gene encoding cytochrome bc1 complex subunit (CR7) in *lses1* exhibited increased protein and transcript abundance, respectively (Table S7). Besides, chlorophyll metabolism-associated genes, such as the genes encoding chloroplastic protein STAY-GREEN (SGR), putative red chlorophyll catabolite reductase (rccR) and ABC transporter C (ABCC3) in *lses1* also showed higher transcription levels than those in WT (Table S7). Taken together, the transcription or abundance of these genes/proteins increased and decreased, revealing changes in chlorophyll metabolism and redox homeostasis in *lses1* leaves, which was basically consistent with the previous physiological characteristic detection results.

### Multiple metabolic regulation mediated by differentially abundant genes/proteins

**Fig. 6:**
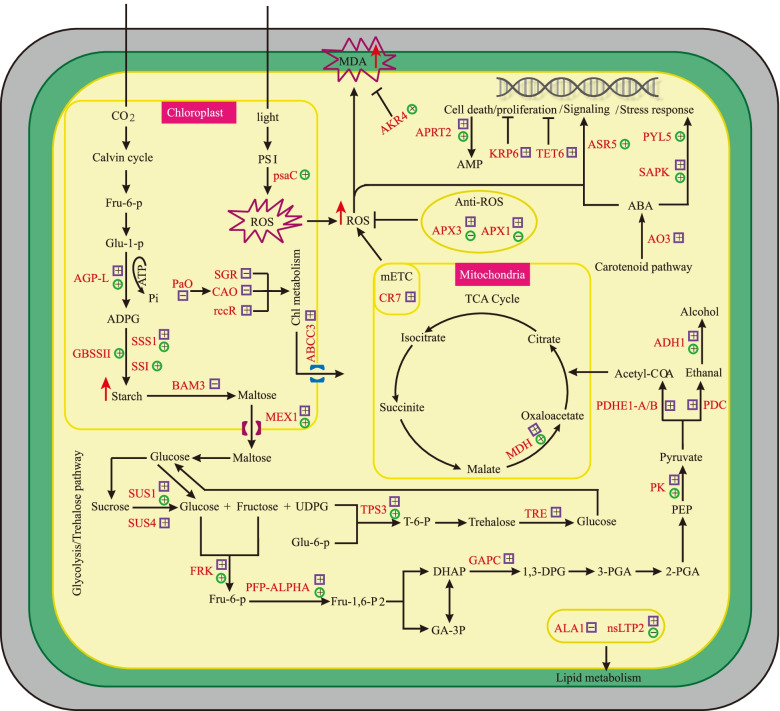
Schematic presentation of metabolic regulation mediated by differentially abundant genes/proteins in the *lses1* mutant (not all the identified genes/proteins are included). The genes/proteins marked red were regulated in *lses1*, purple square and green circle indicate transcript and protein abundance, respectively, “+” represents upregulation, “-” represents downregulation. AGP-L: glucose-1-phosphate adenylyltransferase large subunit; SSI: chloroplastic starch synthase; GBSSII: chloroplastic granule-bound starch synthase II; SSS1: chloroplastic soluble starch synthase 1; BAM3: chloroplastic β-amylase 3; MEX1: chloroplastic maltose excess protein 1-like; SUS: sucrose synthase; FRK: fructokinase-2; PFP-ALPHA: pyrophosphate--fructose 6-phosphate 1-phosphotransferase subunit alpha; GAPC: glyceraldehyde-3-phosphate dehydrogenase 1; PDC: pyruvate decarboxylase; PDHE1-A/B: pyruvate dehydrogenase E1 component subunit; PK: cytosolic pyruvate kinase isozyme; ADH1: alcohol dehydrogenase 1; MDH: malate dehydrogenase; TPS3: trehalose-6-phosphate synthase 3; TRE: trehalase; psaC: photosystem I iron-sulfur centre; CR7: cytochrome bc1 complex subunit; APX: L-ascorbate peroxidase; AKR4: aldo-keto reductase; SGR: chloroplastic stay-green protein; CAO: chloroplastic chlorophyllide a oxygenase; rccR: putative red chlorophyll catabolite reductase; PaO: pheophorbide a oxygenase; ABCC3: ABC_transporter; ALA1: phospholipid-transporting ATPase 1; nsLTP2: non-specific lipid-transfer protein 2; AO3: putative aldehyde oxidase; APRT: adenine phosphoribosyltransferase form 2; KPR6: cyclin-dependent kinase inhibitor 6; TET6: tetraspanin-6; ASR5: abscisic stress-ripening protein 5; PYL5: ABA receptor 5; SAPK3: serine/threonine-protein kinase; ADPG: adenosine diphosphate glucose; UDPG: uridine diphosphate glucose; DHAP: dihydroxyacetone phosphate; 1,3-DPG: 1,3-diphosphoglycerate; 3-PGA: 3-phosphoglycerate; 2-PGA: 2-phosphoglycerate; PEP: phosphoenolpyruvate; AMP: adenosine monophosphate. “→” indicates positive regulation, “-|” represents inhibition, and red “↑” indicates increased content of metabolites determined by biochemical assays

A possible schematic for the metabolic regulation mediated by partial differentially abundant genes/proteins was provided to explain the changes in multiple metabolic levels in the leaves of *lses1*. As shown in Fig. 6 and Table S8, 35 genes were used to describe the pathways related to carbohydrate metabolism, redox homeostasis and cell signaling in *lses1* leaves, and the changes in transcription and protein levels of each gene were marked. The results implied that the excessive accumulation of temporary starch might be the main factor of premature senescence and imbalance of carbohydrate metabolism in *lses1* leaves, and the mutant gene *LSES1/OsCKI1* may regulate starch metabolism and cell apoptosis through ABA-mediated signaling.

## Discussion

### Premature senescence and excess starch phenotype induced by *LSES1* mutation

Assimilate partitioning has long been recognized as a target for crop improvement because it can limit the yield potential of crop plants. A phenotype involving an excessive accumulation of starch and growth retardation is often observed when any inhibition in the export of photoassimilate or in starch metabolism occurs in the leaves [28]. In this study, the *OsCKI1*-deficient allelic mutant *lses1* showed obvious premature senescence and an excess starch phenotype in leaves. Compared with WT, older leaf blades in *lses1* displayed chlorosis at the tip from the third-leaf stage, and the chlorosis degree deepened with the development process and gradually developed to higher leaf positions (Fig. 1-A). In particular, the starch content and I_2_-KI staining analysis indicated that *lses1* presented an abnormal starch accumulation phenotype in leaves during premature senescence (Fig. 1-B and Table S1). This phenotype was similar to that of three reported rice mutants, *esl10* [29], *ossac3* [30] and *ossac4* [31]. Although *LSES1* is allelic to *OsCKI1*/*hbd2*/*LTRPK1*/*LTG1* and the mutation site in the *lses1* mutant is similar to that in the *ltg1* mutant (our unpublished data), no results have been reported on the premature senescence and excess starch phenotype in the leaves of *OsCKI1*-deficient allelic mutants except *lses1*. Therefore, the *lses1* mutant is an ideal material for studying the molecular mechanism of premature leaf senescence and temporary starch metabolism via *LSES1/OsCKI1* and the pleiotropism of *LSES1/OsCKI1* in rice.

### Global changes in the transcriptome and proteome in *lses1*

To comprehensively explore the mechanism of premature leaf senescence caused by temporary starch regulation and the changes in metabolic levels during senescence, transcriptome and proteome analyses were conducted, and the results showed global changes in mRNA and protein levels in *lses1* leaves (Fig. 2-C and Fig. 3-B). Furthermore, subsequent functional analysis of the 4989 differentially expressed genes (DEGs) and 568 differentially expressed proteins (DEPs) showed that the DEGs were mainly involved in carbohydrate metabolic process, oxidation reduction and response to abiotic stimulus (Fig. 2-D), while the DEPs were heavily involved in translation, cytoplasmic part and biosynthetic process (Fig. 3-C). Gene expression is usually affected by transcription, posttranscriptional regulation, RNA splicing, translation, and posttranslational modification, which results in changes in mRNA and protein levels that are not always the same. In this study, approximately 20% of the identified proteins were also found to be regulated at the transcriptional level. The congruence between proteomics and transcriptome data seems to be very poor, which is consistent with general observations [7, 8, 27, 32, 33].

### Metabolic imbalance induced an excessive accumulation of starch and affected glycolysis/TCA cycle in *lses1* leaves

The metabolism of temporary starch may be a complex network formed by the coordinated expression of numerous genes. In this study, several key proteins involved in starch biosynthesis, such as AGP-L (glucose-1-phosphate adenylyltransferase large subunit), GBSSII (granule-bound starch synthase II), SSS1 (chloroplastic soluble starch synthase 1) and SSI (chloroplastic starch synthase I), all exhibited increased abundance in *lses1* (Fig. 6). Notably, AGP-L is the first rate-limiting enzyme in starch biosynthesis, and its overexpression rice plants show significantly increased starch content in leaves [34]. Moreover, several β-amylases in *lses1* showed down-regulated transcription levels. A striking sample is BAM3 (Fig. 6), which encodes the most important β-amylase that strongly participates in the decomposition of temporary starch in leaves at night, and its functional deletion mutant exhibits a higher transitory starch accumulation phenotype in leaves than does other β-amylase deletion mutants [35]. Temporary starch accumulated in the chloroplast during the day and broken down into maltose or glucose at night to maintain the normal growth and development of plants [36, 37]. Excessive accumulation of temporary starch will directly destroy the structure and functioning of chloroplast, leading to low photosynthetic efficiency, ROS production and ultimately to premature leaf senescence [29–31], which might be an important factor in the premature senescence of *lses1* mutant leaves.

Furthermore, sugar as an important signal plays a vital role in controlling plant growth and development [38]. Impaired temporary starch degradation will firstly lead to sugar starvation, subsequently to carbohydrate metabolic disorders, finally to premature leaf senescence [19, 39]. In this study, the expression levels of multiple genes/proteins related to carbohydrate metabolism downstream of starch metabolism in *lses1* were almost all upregulated (Fig. 6). SUS (sucrose synthase 1) overexpression provides more UDP-glucose and fructose for various metabolic pathways [40], while FRK (fructokinase-2) upregulation catalyses the formation of more fructose-6-phosphate derived from fructose and channels sucrose into the glycolytic pathway [41]. It could be speculated that the substrate of glycolysis is more biased towards fructose and sucrose rather than glucose derived from starch due to the accumulation of starch in the chloroplast reduces the transport of maltose and glucose to the cytoplasm. The increased intermediate products further induce the upregulation of downstream glycolysis/TCA cycle-related genes (Fig. 6). Interestingly, sugar starvation caused by incomplete breakdown of temporary starch may enhance the substrate competitiveness of downstream sugar metabolism-related pathways. In cluster 3 of PPI network (Fig. 4A), PFK (ATP-dependent 6-phosphofructokinase) and G6PD (glucose-6-phosphate 1-dehydrogenase) were up-regulated in *lses1* mutant and found to interact, which is due to the connection of substrates between glycolysis and the pentose phosphate pathway. On the other hand, the additional sucrose consumption induces the upregulation of MEX1 (chloroplastic maltose excess protein 1-like) and TRE (trehalase) to maintain a stable sucrose content (Fig. 6), which is possible a strategy in respond to sugar starvation. MEX1 is the main carrier of maltose from chloroplasts to the cytoplasm for sucrose synthesis [42], while TRE is involved in trehalose metabolism, playing an important role in regulating the balance of sucrose and trehalose contents [43].

Taken together, these results implied that the metabolic imbalance of transitory starch, which appeared as increased starch biosynthesis and incomplete starch degradation, which coincided with the abovementioned phenotype and physiological characteristics in *lses1*. The excessive accumulation of starch in *lses1* leaves might lead firstly to chloroplast damage and sugar starvation, subsequently to sugar metabolism disorder, finally to premature senescence.

### Increased premature senescence-related metabolism in *lses1*

Comparative transcriptomic and proteomic analyses implied that in addition to carbohydrate metabolism-related genes/proteins, other mechanisms might contribute to explaining the premature senescence phenotype of *lses1* mutant leaves. The leaves of *lses1* presented increased ROS and MDA contents compared to WT (Fig. 1-C top). These physiological differences were also reflected in the changes in mRNA and protein levels. For example, the protein psaC (photosystem I iron-sulfur centre) and gene CR7 (cytochrome bc1 complex subunit, complex III) in *lses1* showed increased protein and transcript abundance, respectively (Fig. 6). Among them, psaC is involved in ROS generation in the chloroplastic electron transport chain [44], while CR7 affects the electron transfer rate of the mitochondrial respiratory chain [45]. The leaves of *lses1* showed lower SOD, CAT and POD activities compared with WT (Fig. 1-C bottom-left). Interestingly, several L-ascorbate peroxidases, such as APX1 and APX3, in *lses1* all exhibited increased transcripts and decreased protein abundance (Fig. 6), which suggested that the expression of APXs is posttranscriptionally regulated [46]. The contents of chlorophyll a, chlorophyll b and total photosynthetic pigment in *lses1* leaves were significantly lower than those in WT (Fig. 1-C bottom-right). As expected, several photosynthetic pigment anabolism-associated genes in *lses1* were regulated. For example, CAO (chlorophyllide *a* oxygenase), which is involved in chlorophyll *b* biosynthesis [47], and rccR (putative red chlorophyll catabolite reductase), which is involved in chlorophyll *a* degradation [48], showed down- and up-regulated transcription levels, respectively (Fig. 6). SGR (protein stay-green) triggers chlorophyll degradation in natural or dark-induced leaf senescence [49], and its overexpression will increase the degradation of chlorophyll in rice leaves [50]. Interestingly, SGR in *lses1* mutant showed reduced transcription (Fig. 5), which may be due to PaO (pheophorbide a oxygenase) downregulation (FC = 0.66) negatively effects SGR expression [51, 52]. Rice PaO deficiency can also affect the expression of CAO and rccR, accumulate O_2_-, and increase chlorophyll degradation [52], which is consistent with that observed in *lses1* mutant (Fig. 6; Fig. 1C). Moreover, in cluster 4 of PPI network (Fig. 4A), two phospholipid metabolism-related proteins were found to interact: PSD1 (phosphatidylserine decarboxylase proenzyme 1) and PLD1 (phospholipase D). PSD1 and PLD1 are respectively involved in the synthesis and decomposition of NAPEs (N-acylphosphatidylethanolamines) [53, 54], which exist during cell damage [55].

In brief, the results showed increased premature senescence-related metabolism in *lses1* and suggested consistency between the transcriptomic and proteomic analyses and the physiological determination of lipid peroxidation and ROS-scavenging enzymes.

### Increased nucleotide degradation and inhibited cell proliferation in *lses1*

Senescence is considered a self-saving strategy in plants, during which plants recycle and deliver nutrients from degraded proteins, lipids, and nucleic acids to still growing sites [56]. A similar senescence-induced plant adaptation also occurred in the *lses1* mutant described herein. In this study, APRT (adenine phosphoribosyltransferase form 2) in *lses1* showed a higher protein abundance level than that in WT (Fig. 6); this gene is involved in step 1 of the subpathway in the salvage pathway that synthesizes AMP (adenosine monophosphate) from degraded adenine [57]. In addition to DNA damage, plant cell cycle progression and differentiation are closely related to cell death [58]. Interestingly, TET6 (tetraspanin-6), which is considered a senescence-associated protein and is involved in leaf and root growth via negative regulation of cell proliferation in *Arabidopsis* [59], was found to be expressed in *lses1* but not in WT (Fig. 6). Additionally, KRP6 (cyclin-dependent kinase inhibitor 6) was found to be overexpressed in *lses1* mutant (Fig. 6), which accumulates in *Arabidopsis* lacking CK1s/AELs will inhibit cell proliferation [60] and increase cell death during leaf senescence [58]. In cluster 1 of PPI network, four proteins, eIF3f, eIF3l, eIF3a and eIF3g, encoding the eukaryotic translation initiation factor 3 subunit, were found to interact, which may be due to their functional links in modulating the global translation rate to regulate cell growth, proliferation and differentiation [61]. Taken together, these results implied that increased nucleotide degradation and inhibited cell proliferation exacerbated apoptosis and premature senescence of *lses1* mutant leaves.

### ABA may involve in multiple metabolic regulation via *LSES1*/*OsCKI1*

The rapid accumulation of ABA is considered one of the key characteristics of plants in response to abiotic stress [62], and it is related to the changes in starch metabolism induced by stress [63]. In this study, four proteins associated with ABA accumulation and signaling, including ASR5 (abscisic stress-ripening protein 5), ASR2 (abscisic stress-ripening protein 2 fragment), PYL5 (ABA receptor 5) and SAPK (serine/threonine-protein kinase), showed significantly increased abundances in the *lses1* mutant (Fig. 6). Among them, ASR5 is involved in the ABA-mediated stomatal closure pathway in response to drought and osmotic stress, and rice plants overexpressing ASR5 gene exhibit higher endogenous ABA accumulation [64]. PYL5 functions as a positive regulator of abiotic stress-responsive gene expression [65], which together with SAPK2, is part of an ABA signaling unit that modulates seed germination and early seedling growth [66]. *OsCKI1/hbd2/LTRPK1/LTG1*, which is involved in hybrid breakdown, root development, hormone response, and cold adaptation, has been cloned utilizing multiple rice mutants with different phenotypes [10–13]. Liu et al. (2003) showed that OsCKI1 protein can phosphorylate casein, and may mediate the interaction of ABA and other hormones signaling in rice through hierarchical phosphorylation [11]. CKI1 inhibits gibberellin signaling and rice heading also due to its role of phosphorylation [14]. The phosphorylation substrate diversity of CKI1 determines its important roles in plant growth and development. In *Arabidopsis* CKI1 deficiency mutant, the DEG KRP6 we identified was found to be highly expressed due to the weakened phosphorylation of CKI1, leading to cell death and inhibited cell cycle process [58, 60]. Recent study reports that mutation of *Arabidopsis* CK1 causes reduced phosphorylation, ubiquitination, and degradation of ABA receptors PYR/PYLs, thereby enhancing the ABA responses [18], which is similar to the result observed in the *lses1* mutant. In cotton, CKI1 has starch synthase kinase activity, and its overexpression can inactivate AGPase (ADP glucose pyrophosphorylase), SSS (soluble starch synthase) and GBSS (granule-bound starch synthase), causing imbalance in carbohydrate metabolism [19]. Interestingly, AGP-L, GBSSII and SSS1 were up-regulated in *lses1* mutant (Fig. 6). It could be speculated that AGP-L, GBSSII and SSS1 may be potential target genes of CKI1, and reduced phosphorylation enhances the stability of these three starch synthases, which might be the main factor for the excessive accumulation of starch in *lses1* leaves. In addition, multiple primary carbohydrate metabolism-related genes were found to be regulated by ABA in previous transcriptomic and proteomic studies [67–69]. For example, the accumulation of AGP-L transcript is known to cooperatively regulated by ABA and sucrose, and their expression patterns were elevated significantly by the co-treatment of sucrose and ABA [70, 71]. Taken together, these results implied that regulation of post-translational levels is critical to the mechanism of rice leaf starch metabolism, and *LSES1*/*OsCKI1* may affect temporary starch accumulation and premature leaf senescence through crosstalk ABA signaling and starch metabolism pathways.

## Conclusion

The *osckI1* allelic mutant *lses1*, which displayed premature senescence and an excess starch phenotype in leaves, was comprehensively analysed with transcriptomic and proteomic approaches. The differentially expressed genes (DEGs) and proteins (DEPs) revealed changes in several important metabolic mechanisms, such as carbohydrate metabolism, pigment metabolism, redox regulation and cell signaling. A possible schematic for the metabolic regulation mediated by differentially abundant genes/proteins was provided to explain the changes in multiple metabolic levels in the leaves of *lses1* (Fig. 6 and Table S8). The results implied that the excessive accumulation of temporary starch in leaves was the main factor that induced premature senescence of *lses1* leaves and changes in several carbohydrate metabolism levels (including glycolysis/TCA cycle and trehalose metabolism) downstream of starch metabolism. The protein-protein interaction (PPI) network might play an auxiliary role in promoting premature leaf senescence in *lses1*. In addition, the possible potential roles of *LSES1/OsCKI1* gene in cell signaling were discussed. *LSES1/OsCKI1* might participate in the regulation of starch metabolism, cell apoptosis and ABA-mediated signaling, but future studies are required to verify it. Altogether, our study provides novel insight into the metabolic mechanism of temporary starch via *LSES1/OsCKI1* in rice leaves.

## Supplementary Information

**Additional file 1: Table S1.** Detection data of various physiological characteristics in leaves of the wild type (WT) and the *lses1* mutant. **Table S2.** Summary of the transcriptome sequencing quality. **Table S3.** Identification of differentially expressed genes in leaves of the wild type (WT) and the *lses1* mutant. **Table S4.** Identification of differentially expressed proteins in leaves of the wild type (WT) and the *lses1* mutant. **Table S5.** Protein-protein interactions of the four functional clusters. **Table S6.** List of partial differentially expressed genes/proteins related to carbohydrate metabolism. **Table S7.** List of partial differentially expressed genes/proteins involved in apoptosis-related metabolism. **Table S8.** List of differentially expressed genes/proteins involved in multiple metabolic regulation in schematic.

## Data Availability

All data generated or analysed during this study are included in this published article and its supplementary information files. The sequencing data associated with transcription profiles in this study have been deposited in NCBI SRA database with accession number PRJNA757395 (https://www.ncbi.nlm.nih.gov/sra/PRJNA757395). The proteomics data have been upload in ProteomeXchange database with accession number PXD030669 (http://www.ebi.ac.uk/pride/archive/projects/PXD030669).

## Abbreviations

ABA: Abscisic acid
AMP: Adenosine monophosphate
CAT: Catalase
Chl: Chlorophyll
DEGs: Differentially expressed genes
DEPs: Differentially expressed proteins
ED: End of day
EN: End of night
FDR: False Discovery Rate
GO: Gene Ontology
GO-BP: Gene ontology of biological process
iTRAQ: Isobaric tags for relative and absolute quantification
MDA: Malondialdehyde
MT: Mutant
NAPEs: N-acylphosphatidylethanolamines
PCD: Programmed cell death
POD: Peroxidase
PPI: Protein-protein interaction
PTMs: Posttranslational modifications
ROS: Reactive oxygen species
SOD: Superoxide dismutase
WT: Wild-type variety

## References

[1] Geigenberger P (2011). Regulation of starch biosynthesis in response to a fluctuating enviorenment. Plant Physiol.

[2] Lloyd JR, Kossmann J (2015). Transitory and storage starch metabolism: two sides of the same coin?. Curr Opin Biotech.

[3] Mahlow S, Orzechowsk S, Fettke J (2016). Starch phosphorylation: insights and perspectives. Cell Mol Life Sci.

[4] Latterich M, Abramovitz B, Leyland J (2008). Proteomics: new technologies and clinical applications. Eur J Cancer.

[5] Wilm M (2009). Quantitative proteomics in biological research. Proteomics..

[6] Mann N, Kulak A, Nagaraj N, Cox J (2013). The coming age of complete, accurate, and ubiquitous proteomes. Mol Cell.

[7] Greenbaum D, Colangelo C, Williams K, Gerstein M (2003). Comparing protein abundance and mRNA expression levels on a genomic scale. Genome Biol.

[8] Xiong Q, Feng J, Li ST, Zhang GY, Qiao ZX, Chen Z, Wu Y, Lin Y, Li T, Ge F, Zhao JD (2015). Integrated transcriptomic and proteomic analysis of the global response of Synechococcus to high light stress. Mol Cell Proteomics.

[9] Ma QF, Wu CH, Wu M, Pei WF, Li XL, Wang WK, Zhang JF, Yu JW, Yu SX (2016). Integrative transcriptome, proteome, phosphoproteome and genetic mapping reveals new aspects in a fiberless mutant of cotton. Scientific Rep-uk.

[10] Yamamoto E, Takashi T, Morinaka Y, Lin SY, Wu JZ, Matsumoto T, Kitano H, Matsuoka M, Ashikari M (2010). Gain of deleterious function causes an autoimmune response and Bateson-Dobzhansky-Muller incompatibility in rice. Mol Gen Genomics.

[11] Liu W, Xu ZH, Luo D, Xue HW (2003). Roles of *OsCKI1*, a rice casein kinase I, in root development and plant hormone sensitivity. Plant J.

[12] Liu W, Ji SX, Fang XL, Wang QG, Li Z, Yao FY, Hou L, Dai SJ (2013). Protein kinase LTRPK1 influences cold adaptation and microtubule stability in rice. J Plant Growth Regul.

[13] Lu GW, Wu FQ, Wu WX, Wang HJ, Zheng XM, Zhang YH, Chen XL, Zhou KN, Jin MN, Cheng ZJ, Li XY, Jiang L, Wang HY, Wan JM (2014). Rice LTG1 is involved in adaptive growth and fitness under low ambient temperature. Plant J.

[14] Dai C, Xue HW (2010). Rice early flowering1, a CKI, phosphorylates DELLA protein SLR1 to negatively regulate gibberellin signaling. EMBO J.

[15] Qu L, Wei Z, Chen HH, Liu T, Liao K, Xue HW (2021). Plant casein kinases phosphorylate and destabilize a cyclin-dependent kinase inhibitor to promote cell division. Plant Physiol.

[16] Ben-Nissan G, Cui W, Kim DJ, Yang YD, Yoo BC, Lee JY (2008). Arabidopsis casein kinase 1-like 6 contains a microtubule binding domain and affects the organization of cortical microtubules. Plant Physiol.

[17] Tan ST, Dai C, Liu HT, Xue HW (2013). Arabidopsis casein kinase1 proteins CK1.3 and CK1.4 phosphorylate cryptochrome2 to regulate blue light signaling. Plant Cell.

[18] Chen HH, Li Q, Xu ZH, Zhu JK, Xue HW (2018). EL1-like casein kinases suppress ABA signaling and responses by phosphorylating and destabilizing the ABA receptors PYR/PYLs in *Arabidopsis*. Mol Plant.

[19] Min L, Zhu L, Tu L, Deng F, Yuan D, Zhang X (2013). Cotton *GhCKI* disrupts normal male reproduction by delaying tapetum programmed cell death via inactivating starch synthase. Plant J.

[20] Hagen SR, Muneta P, Tourneau DL, Brown J (2008). Effect of temperature on the starch content of potato callus tissue. Am J Potato Res.

[21] Wellburn AR (1994). The spectral determination of chlorophylls a and b as well as total carotenoids using various solvents with spectrophotometers of different resolution. Plant Physiol.

[22] Han C, He DL, Li M, Yang PF (2014). In-depth proteomic analysis of rice embryo reveals its important roles in seed germination. Plant Cell Physiol..

[23] Anders S, Huber W (2010). Differential expression analysis for sequence count data. Genome Biol.

[24] Guan DH, Tian HL (2017). Integrated network analysis to explore the key genes regulated by parathyroid hormone receptor 1 in osteosarcoma. World J Surg Onc.

[25] Maere S, Heymans K, Kuiper M (2005). BiNGO: a Cytoscape plugin to assess overrepresentation of gene ontology categories in biological networks. BMC Bioinformatics..

[26] Bader GD, Hogue CW (2003). An automated method for finding molecular complexes in large protein interaction networks. BMC Bioinformatics.

[27] Soto-Suárez M, Serrato AJ, Rojas-González JA, Bautista R, Sahrawy M (2016). Transcriptomic and proteomic approach to identify differentially expressed genes and proteins in Arabidopsis thaliana mutants lacking chloroplastic 1 and cytosolic FBPases reveals several levels of metabolic regulation. BMC Plant Biol.

[28] Hirose T, Aoki N, Harada Y, Masaki O, Yoichi H, Ryu O, Miyao A, Hirohiko H, Tomio T (2013). Disruption of a rice gene for α-glucan water dikinase, *OsGWD1*, leads to hyperaccumulation of starch in leaves but exhibits limited effects on growth. Front Plant Sci.

[29] Chen XL, Zhu MD, Gu FX, Liu MM, Zhang YY, Xing YD, Du D, Xiao YH, Zhu XY, He GH (2018). Identification and gene fine mapping of starch accumulation and early senescent leaf mutant esl10 in rice. Crop Sci.

[30] Huang JY, Yan M, Zhu XY, Zhang T, Shen WQ, Yu P, Wang YT, Sang XC, Yu GL, Zhao BB, He GH (2018). Gene mapping of starch accumulation and premature leaf senescence in the *ossac3* mutant of rice. Euphytica..

[31] Zhu MD, Chen XL, Zhu XY, Xing YD, Du D, Zhang YY, Liu MM, Zhang QL, Lu X, Peng SS, He GH, Zhang TQ (2020). Identification and gene mapping of the starch accumulation and premature leaf senescence mutant ossac4 in rice. J Integr Agr.

[32] Gygi SP, Rochon Y, Franza BR, Aebersold R (1999). Correlation between protein and mRNA abundance in yeast. Mol Cell Biol.

[33] Pandey A, Mann M (2000). Proteomics to study genes and genomes. Nature..

[34] Oiestad AJ, Martin JM, Giroux MJ (2019). Yield increases resulting from AGPase overexpression in rice are reliant on plant nutritional status. Plant Growth Regul.

[35] Fulton DC, Stettler M, Mettler T, Vaughan CK, Li J, Francisco P, Gil M, Reinhold H, Eicke S, Messerli G, Dorken G, Halliday K, Smith AM, Smith SM, Zeeman SC (2008). Beta-amylase4, a noncatalytic protein required for starch breakdown, acts upstream of three active beta-amylases in Arabidopsis chloroplasts. Plant Cell.

[36] Streb S, Zeeman SC. Starch metabolism in Arabidopsis. Arabidopsis Book. 2012;10:e0160. 10.1199/tab.0160 PMC3527087 23393426

[37] Ruan YL (2014). Sucrose metabolism: gateway to diverse carbon use and sugar signaling. Annu Rev Plant Biol.

[38] Lastdrager J, Hanson J, Smeekens S (2014). Sugar signals and the control of plant growth and development. J Exp Bot.

[39] Xiao L, Jiang S, Huang P, Chen F, Wang X, Cheng Z, Miao Y, Liu L, Searle I, Liu C, Wu XX, Fu YF, Chen Q, Zhang XM (2020). Two Nucleoporin98 homologous genes jointly participate in the regulation of starch degradation to repress senescence in Arabidopsis. BMC Plant Biol.

[40] Wang AY, Man-Han K, Yang WH, Sayion Y, Liu LF, Lee PD, Su JC (1999). Differentially and developmentally regulated expression of three rice sucrose synthase genes. Plant Cell Physiol..

[41] Šimon M, Shen ZJ, Ghoto K, Chen J, Liu X, Gao GF, Kokalj AJ, Novak S, Drašler B, Zhang JY, You YP, Drobne D, Zheng HL (2020). Proteomic investigation of Zn-challenged rice roots reveals adverse effects and root physiological adaptation. Plant Soil.

[42] Niittylä T, Messerli G, Trevisan M, Chen J, Smith AM, Zeeman SC (2004). A previously unknown maltose transporter essential for starch degradation in leaves. Science..

[43] Müller J, Aeschbacher RA, Wingler A, Boller T, Wiemken A (2001). Trehalose and trehalase in Arabidopsis. Plant Physiol.

[44] Kozi A (1999). The water-water cycle in chloroplasts: scavenging of active oxygens and dissipation of excess photons. Annu Rev Plant Phys.

[45] Zeng DY, Cui J, Yin YS, Zang M, Shan S, Gao X, Zhang YC, Sun YQ, Lu WH (2020). Effects of space flight on expression of key proteins in rice leaves. Rice Sci.

[46] Yoshimura K, Yabuta Y, Tamoi M, Ishikawa T, Shigeoka S (1999). Alternatively spliced mRNA variants of chloroplast ascorbate peroxidase isoenzymes in spinach leaves. Biochem J.

[47] Oster U, Tanaka R, Tanaka A, Rüdiger W (2000). Cloning and functional expression of the gene encoding the key enzyme for chlorophyll b biosynthesis (CAO) from Arabidopsis thaliana. Plant J.

[48] Rodoni S, Vicentini F, Schellenberg M, Matile P, Hortensteiner S (1997). Partial purification and characterization of red chlorophyll catabolite reductase, a stroma protein involved in chlorophyll breakdown. Plant Physiol.

[49] Sakuraba Y, Schelbert S, Park SY, Han SH, Lee BD, Andrès CB, Kessler F, Hörtensteiner S, Paek NC (2012). STAY-GREEN and chlorophyll catabolic enzymes interact at light-harvesting complex II for chlorophyll detoxification during leaf senescence in Arabidopsis. Plant Cell.

[50] Jiang HW, Li MR, Liang NT, Yan HB, Wei YB, Xu XL, Liu J, Xu ZF, Chen F, Wu GJ (2007). Molecular cloning and function analysis of the stay green gene in rice. Plant J.

[51] Park SY, Yu JW, Park JS, Li J, Yoo SC, Lee NY, Lee SK, Jeong SW, Seo HS, Koh HJ, Jeon JS, Park YI, Paek NC (2007). The senescence-induced staygreen protein regulates chlorophyll degradation. Plant Cell.

[52] Zhang Z, He Y, Li L, Zhang X, Xu X, Shi Y, Wu JL (2021). Characterization of a novel allele encoding pheophorbide a oxygenase in rice. Plant Signal Behav.

[53] Nerlich A, Orlow von M, Rontein D, Hanson AD, Dörmann P (2007). Deficiency in phosphatidylserine decarboxylase activity in the psd1 psd2 psd3 triple mutant of *Arabidopsis* affects phosphatidylethanolamine accumulation in mitochondria. Plant Physiol.

[54] Kilaru A, Chapman KD (2012). N-Acylated phospholipid metabolism and seedling growth: insights from lipidomics studies in *Arabidopsis*. Plant Signal Behav.

[55] Lafrance CP, Blochet JE, Pézolet M (1997). N-acylphosphatidylethanolamines: effect of the N-acyl chain length on its orientation. Biophys J.

[56] Nir S, María MRW, Kamolchanok U, Eduardo B (2018). Stress-induced senescence and plant tolerance to abiotic stress. J Exp Bot.

[57] Xing QH, Ru ZG, Li J, Zhou CJ, Jin DM, Sun Y, Wang B (2005). Cloning a second form of adenine phosphoribosyl transferase gene (*TaAPT2*) from wheat and analysis of its association with thermo-sensitive genic male sterility (TGMS). Plant Sci.

[58] Schnittger A, Weinl C, Bouyer D, Schöbinger U, Hülskamp M (2003). Misexpression of the cyclin-dependent kinase inhibitor ICK1/KRP1 in single-celled Arabidopsis trichomes reduces endoreduplication and cell size and induces cell death. Plant Cell.

[59] Wang F, Muto A, Van de Velde J (2015). Functional analysis of the Arabidopsis TETRASPANIN gene family in plant growth and development. Plant Physiol.

[60] Li Q, Zhuang W, Chen HH, Liu T, Liao K, Xue HW. Plant casein kinases phosphorylate and destabilize a cyclin-dependent kinase inhibitor to promote cell division. Plant Physiol. 2021;187(2):917-30. 10.1093/plphys/kiab284 PMC8491028 34608955

[61] Raabe K, Honys D, Michailidis C (2019). The role of eukaryotic initiation factor 3 in plant translation regulation. Plant Physiol Biochem.

[62] Christmann A, Moes D, Himmelbach A, Yang Y, Tang Y, Grill E (2006). Integration of abscisic acid signaling into plant responses. Plant Biol.

[63] Kempa S, Krasensky J, Dal Santo S, Kopka J, Jonak C (2008). A central role of abscisic acid in stress-regulated carbohydrate metabolism. PLoS One.

[64] Li J, Li Y, Yin Z, Jiang JH, Zhang MH, Guo X, Ye JZ, Zhao Y, Xiong HY, Zhang ZY, Shao YZ, Jiang CH, Zhang HL, An G, Paek NC, Ali J, Li ZC (2017). *OsASR5* enhances drought tolerance through a stomatal closure pathway associated with ABA and H_2_O_2_ signaling in rice. Plant Biotechnol J.

[65] Kim H, Lee K, Hwang H, Bhatnagar N, Kim DY, Yoon IS, Byun MO, Kim ST, Jung KH, Kim BG (2014). Overexpression of PYL5 in rice enhances drought tolerance, inhibits growth, and modulates gene expression. J Exp Bot.

[66] Kim H, Hwang H, Hong JW, Lee YN, Ahn IP, Yoon IS, Yoo SD, Lee S, Lee SC, Kim BG (2012). A rice orthologue of the ABA receptor, OsPYL/RCAR5, is a positive regulator of the ABA signal transduction pathway in seed germination and early seedling growth. J Exp Bot.

[67] Choudhury A, Lahiri A (2011). Comparative analysis of abscisic acid-regulated transcriptomes in *Arabidopsis*. Plant Biol.

[68] Huang YQ, Cai SG, Zeng JB, Wu DZ, Zhang GP (2017). Isobaric tags for relative and absolute quantitation proteomic analysis of germinating barley under gibberellin and abscisic acid treatments. J Agric Food Chem.

[69] Yoshida T, Fujita Y, Maruyama K, Mogami J, Todaka D, Shinozaki K, Yamaguchi-Shinozaki K (2014). Four *Arabidopsis* AREB/ABF transcription factors function predominantly in gene expression downstream of SnRK2 kinases in abscisic acid signaling in response to osmotic stress. Plant Cell Enviro.

[70] Akihiro T, Mizuno K, Fujimura T (2010). Gene expression of ADP-glucose pyrophosphorylase and starch contents in rice cultured cells are cooperatively regulated by sucrose and ABA. Plant Cell Physiol.

[71] Li YP, Yu GW, Lv YA, Long TD, Li P, Hu YF, Liu HM, Zhang JJ, Liu YH, Li WC, Huang YB (2018). Combinatorial interaction of two adjacent cis-active promoter regions mediates the synergistic induction of Bt2 gene by sucrose and ABA in maize endosperm. Plant Sci.

